# Homoeologs in Allopolyploids: Navigating Redundancy as Both an Evolutionary Opportunity and a Technical Challenge—A Transcriptomics Perspective

**DOI:** 10.3390/genes15080977

**Published:** 2024-07-24

**Authors:** Gaetano Aufiero, Carmine Fruggiero, Davide D’Angelo, Nunzio D’Agostino

**Affiliations:** Department of Agricultural Sciences, University of Naples Federico II, 80055 Portici, Italy; gaetano.aufiero@unina.it (G.A.); carmine.fruggiero@unina.it (C.F.); davide.dangelo@unina.it (D.D.)

**Keywords:** polyploids, homeologs, allopolyploidization, RNA-sequencing, gene expression, expression level dominance, homoeolog expression bias, additivity, bioinformatics

## Abstract

Allopolyploidy in plants involves the merging of two or more distinct parental genomes into a single nucleus, a significant evolutionary process in the plant kingdom. Transcriptomic analysis provides invaluable insights into allopolyploid plants by elucidating the fate of duplicated genes, revealing evolutionary novelties and uncovering their environmental adaptations. By examining gene expression profiles, scientists can discern how duplicated genes have evolved to acquire new functions or regulatory roles. This process often leads to the development of novel traits and adaptive strategies that allopolyploid plants leverage to thrive in diverse ecological niches. Understanding these molecular mechanisms not only enhances our appreciation of the genetic complexity underlying allopolyploidy but also underscores their importance in agriculture and ecosystem resilience. However, transcriptome profiling is challenging due to genomic redundancy, which is further complicated by the presence of multiple chromosomes sets and the variations among homoeologs and allelic genes. Prior to transcriptome analysis, sub-genome phasing and homoeology inference are essential for obtaining a comprehensive view of gene expression. This review aims to clarify the terminology in this field, identify the most challenging aspects of transcriptome analysis, explain their inherent difficulties, and suggest reliable analytic strategies. Furthermore, bulk RNA-seq is highlighted as a primary method for studying allopolyploid gene expression, focusing on critical steps like read mapping and normalization in differential gene expression analysis. This approach effectively captures gene expression from both parental genomes, facilitating a comprehensive analysis of their combined profiles. Its sensitivity in detecting low-abundance transcripts allows for subtle differences between parental genomes to be identified, crucial for understanding regulatory dynamics and gene expression balance in allopolyploids.

## 1. Introduction

Allopolyploids play a critical role in plant evolution. By combining the genomes of different species, they contribute to genetic diversity and adaptation, which can result in new species with enhanced survival capabilities [1]. This genetic amalgamation often leads to increased heterozygosity, providing a buffer against environmental stresses and diseases [1]. Additionally, allopolyploids can exhibit novel traits not present in their parent species, driving evolutionary innovation. For example, the evolution of common/bread wheat (*Triticum aestivum*) showcases how hybridization and polyploidy can result in a more robust species with significantly improved agronomic traits, such as tolerance to salt, low pH, and better resistance to several pests and diseases [2]. The economic impact of allopolyploids is significant, especially in agriculture and horticulture. Numerous staple crops and commercially valuable plants are allopolyploids, including key cash crops like wheat, cotton, and canola. Global wheat production exceeds 780 million tonnes [3], with approximately 95% of this yield coming from the hexaploid species *T. aestivum* [2]. Cotton (*Gossypium hirsutum* and *Gossypium barbadense*) is commercially important for its fibres that possess both increased quality and yield compared to their progenitors [4]. Another example is canola (*Brassica napus*), which, unlike its parents (*Brassica rapa* and *Brassica oleracea*), is economically important as an oil crop [5]. These examples underscore how allopolyploidy enhances traits crucial for economic success in agriculture, benefiting industries worldwide.

Transcriptomics involves studying the complete set of RNA transcripts (i.e., the transcriptome) present in a specific cell or tissue type at a particular developmental stage and/or under specific physiological conditions.

In allopolyploid plants, the transcriptome mirrors the expression of duplicated genomes arising from hybridization. This phenomenon gives rise to intricate gene expression patterns, including homoeolog expression bias, differential usage of homoeologs under specific condition, expression level dominance, and transgressive regulation.

Analysing transcriptomes in allopolyploid plants helps scientists understand the evolution of duplicated genes [6,7,8,9] and how these plants adapt to new environments [10,11]. Additionally, it aids in identifying novel genes and isoforms [12,13,14] that may be important for the characterization of important traits for breeding programs and biotechnological applications, such as resistance to abiotic stress [15,16], tolerance to nutrition deficiency [17], disease resistance [18,19], increased yield [20,21,22], restoration of fertility [22], improvement in fibre quality [23], and biofortification [24].

Transcriptome analysis in allopolyploid species presents numerous bioinformatics challenges due to the coexistence of highly similar gene pairs, known as homoeologs. These genes originated through speciation and were later combined in the same genome by allopolyploidization [25]. These challenges include choosing optimal strategies and parameters for the assembly and mapping of RNA-seq reads, avoiding chimeric assemblies of homoeolog gene copies, distinguishing individual homoeolog expression levels, and detecting novel genes, alternative splicing and polyadenylation events [26,27,28,29,30,31,32,33]. Therefore, transcriptome analysis in allopolyploid is both a valuable and challenging research topic.

## 2. Terminology in Allopolyploid Gene Expression

Recent studies on gene expression in allopolyploids have revealed confusion regarding the terminology used. Clear definitions are essential to avoid misunderstandings and ensure effective scientific communication. This confusion often stems from variations in analytical methods and differences in the target organisms studied.

The term “genomic dominance”, “genomic expression dominance” or “expression dominance” in allopolyploids was originally described by Rapp et al. [34]. They used these terms to describe situations where the total expression level of a gene or group of homoeolog genes equals that of one of the parental genomes. Their study focused on a synthetic allopolyploid (A2D1), obtained from the inter-specific cross between the two diploid species *Gossypium arboreum* (genome A2) and *Gossypium thurberi* (genome D1) followed by chromosomal doubling with colchicine. In this synthetic allopolyploid, they observed that the expression levels of approximately 11,000 genes, both up- and down-regulated, were statistically equivalent to those of the D1 genome. Further studies on genome dominance in allopolyploid cotton were conducted by Flagel and Wendel [35]. The same concept was applied by Bardil et al. [36] to the *Coffea arabica* allopolyploid. Similarly, the term “parental dominance” has been used to describe a condition where the overall gene expression of the allopolyploid mirrors the overall expression level of one parent [37,38,39].

Subsequently, Grover et al. [40] suggested replacing the term “genome dominance” with “expression level dominance”. This refers to the situation where the expression level of a gene in the allopolyploid is closer to the expression level of the same gene in one of the parental species rather than being an average or intermediate of both parents. This change was suggested also to preserve the original meaning while eliminating the ambiguity associated with the word “genome”.

In other studies, the term “genome dominance” or “sub-genome dominance” is used to describe certain phenomena, e.g., lower DNA methylation, higher gene retention and preferential expression, attributable to one of the parental genomes of the allopolyploid [41,42,43,44,45]. Recently, Kopecký et al. [46] defined “genome dominance” in allopolyploids more generally as the situation in which one parental genome becomes dominant over the other. Furthermore, Grover et al. [40] pointed out that “expression level dominance” cannot be detected without measuring the expression level in the two parental genomes. Therefore, it is difficult to calculate this value in paleopolyploids because the parents may have become extinct or have undergone substantial genomic and/or transcriptomic changes [47]. This concept was also highlighted in a review by Buggs et al. [48]. The “expression level dominance” is independent of the relative contribution to expression level of the two homoeologs. In other words, an allopolyploid formed from two parental species (A and B), where gene X has different expression levels (10 in A and 20 in B), may express gene X at the same level as one of the parents (either 10 or 20). This expression level is irrespective of whether the two homoeologs contribute equally or unequally to the total expression of gene X in the allopolyploid.

Currently, several studies have used the term “expression level dominance” as a synonym for the term “genomic dominance” or “genome expression dominance” sensu Rapp et al. [34,45,46,49,50,51,52,53]. “Expression level dominance” could be balanced (the same number of duplicated gene pairs in the allopolyploid have the overall expression level of one of the parental species, e.g., considering 10 duplicated gene pairs in the allopolyploid, the total expression of 5 homoeolog gene pairs is equivalent to one of the parents while the total expression of the remaining 5 homoeolog gene pairs is equivalent to the other parent) or unbalanced (more homoeolog genes than half have the expression level of one of the parental species). 

**Figure 1:**
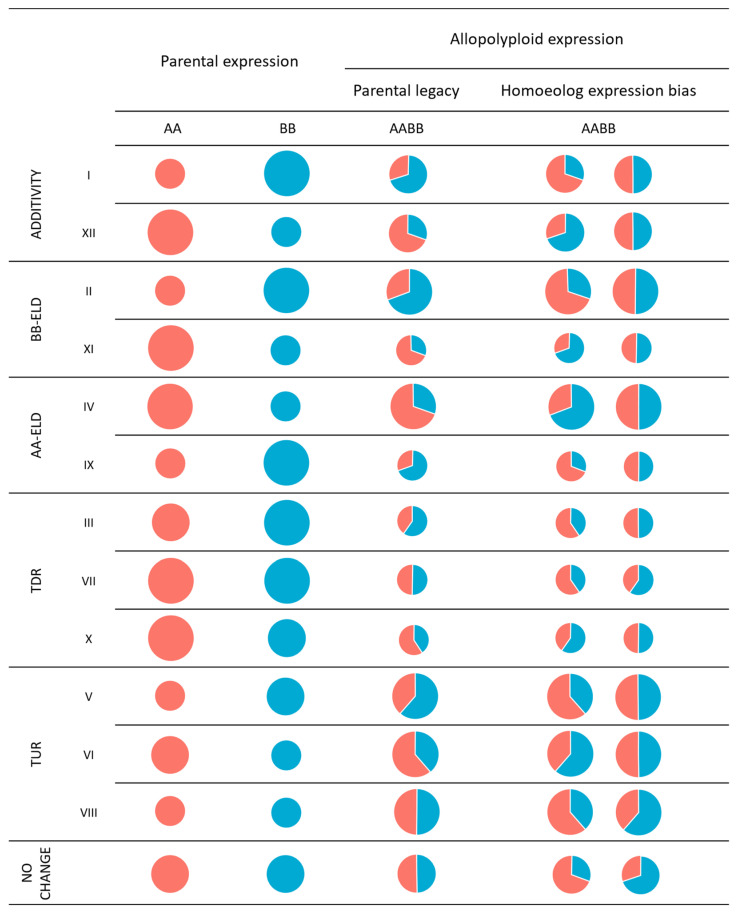
According to Rapp et al. [34], the 12 states in which the differentially expressed genes between allopolyploid and parents are binned are represented by Roman numbers from I to XII. The area of the circle represents the overall expression level of a single gene. Red and blue colours represent the expression traceable to the parental genomes AA and BB, respectively. AA, BB and AABB represent the two diploid parental species and allopolyploid species, respectively. Additivity (first and second rows) is calculated as the mid-parent value (MPV). AA-ELD and BB-ELD (from the third to the sixth rows) represents the expression level dominance in favour of the parental genome AA and BB, respectively, i.e., the overall expression level of both homoeologs, relative to a gene, is equivalent to the overall expression level, for the same gene, of one of the parental species (AA or BB). TDR and TUR (seventh to twelfth) represent transgressive down-regulation and transgressive up-regulation, respectively, i.e., the overall expression level of both homoeologs, relative to a gene, is outside the range of the parental expression level for the same gene. NO CHANGE (thirteenth row) represents the absence of differential expression of genes between parents and the allopolyploid. The relative contribution of homoeolog expression can either proportionally reflect pre-existing differences between the parental species (parental legacy) or show bias towards one of the parental species (homoeolog expression bias, illustrated by two examples per state).

According to Grover et al. [40], the term “homoeolog expression bias” can be used to describe the condition in which a duplicated gene in an allopolyploid is preferentially expressed by one homoeolog, generally resulting in an unequal expression level between the homoeologs (Figure 1). As observed for expression level dominance, homoeolog expression bias could be balanced (there is an equal number of genes preferentially expressed by each homoeolog, e.g., considering 10 duplicated genes, 5 are preferentially expressed by one homoeolog and the remaining 5 are preferentially expressed by the other) or unbalanced (the number of expressed genes favour one homoeolog over other). In other words, if the expression level of a gene is equivalent between the two diploid parents but becomes unequal between the homoeologs in an allopolyploid, then the gene exhibits an expression bias (i.e., novel bias in progeny). Similarly, this bias is also observed if the diploid parents display non-equivalent expression levels while the homoeologs in the allopolyploid show equivalent expression levels [54]. This last case is more commonly described as “no bias in progeny” [50,51,53], whereby an expression bias in the parent is reverted to non-differential expression between homoeologs.

A final scenario occurs when the observed bias between homoeologs reflects the bias found between the corresponding parental genes (i.e., parental legacy). In a practical analysis of homoeolog expression bias, once homoeolog gene pairs are identified in allopolyploids, the expression level of these genes are compared to those of the respective diploid parent using statistical tests such as Fisher’s exact test [49], Student’s *t*-test (*p*  ≤  0.05) [50], Wald test/DESeq2 [51] or by a Poisson distribution test [52]. This approach allows for the classification of homoeolog gene pairs into three categories: those maintaining parental conditions, those showing no bias in progeny, and those exhibiting novel bias in progeny [51].

It can be deduced that to define an expression bias between homoeologs, as in the case of “expression level dominance”, it is necessary to also take into consideration the expression levels of the parental genomes. Therefore, three conditions generally occur (synthetically originated, naturally originated, missing parents). 

In the “synthetically originated” condition, the allopolyploid has been synthetically originated and the expression levels of its parents are available [50]. In this case, there is great interest in studying the phenomena that are generally defined as ‘genome shock’ and ‘transcriptome shock’ [1,55,56]. That is, all of the phenomena of rapid reorganization of the genome and gene expression that occur after hybridization and duplication. 

In the “naturally originated” condition, the allopolyploid originating from the natural hybridization of its two parent species and expression levels of its parents are available [53]. In this case, it is particularly interesting to observe which genetic, genomic, and epigenetic changes have occurred since the hybridization and polyploidization event over a long period. Nevertheless, the great challenge is represented by the identification of the right parentals (i.e., the expression profile of the parental species that generated the allopolyploid). Considering that not only the allopolyploid but also its parents, assuming they still exist, have undergone evolutionary mechanisms, we cannot be certain that the identified parents are truly representative of the original parents, especially when considering polyploidization events that occurred several million years ago [47]. 

In the two conditions described above, if the expression levels of the parents are not equal, the expression bias of the homoeologs has been evaluated by comparing the expression value of each homoeolog with a parental mix expression value [35] or more frequently with the parental expression level estimated separately [50,53].

In a “missing parents” condition, the parental expression levels are not available, but as long as it is possible to identify the parental genomes in the allopolyploid, an equal expression level of the two parental genomes is usually assumed [41,57]. If we assess homoeolog expression bias without knowing the parental expression levels, we cannot dismiss the possibility that the bias between homoeologs is merely a reflection of parental inheritance (i.e., parental legacy). Thus, the expression differences observed between homoeologs might have already been present in the parental species (Figure 1). In other words, for a given gene or group of genes, the relative contribution of expression level between homoeologs in the allopolyploid is equal to the relative contribution of expression level between parents. This situation could be described as an additive expression of homoeologs [48,58]. But generally, the term “additive” describes a situation in which the total expression of both homoeologs in an allopolyploid, for a gene or a group of genes, equals the arithmetic mean, also known as the mid-parent value (MPV) [35,39,49,50,53], or as discussed by Gianinetti [59], the sum of parental species expression (also defined as summed-parent value “SPV”). According to Gianinetti [59], the term “MPV” is used to calculate additivity by assuming that each parent contributes half of their genome to the offspring. However, in the case of neo-polyploidy, where the offspring receives each parent’s entire genome, parental values should be summed, not averaged, if they are truly additive. This is because the entire genome, rather than half, is passed on to offspring. However, MPV is the generally used method to calculate the additive expression value (Figure 1). As discussed by Buggs et al. [48], there are at least two ways in which MPV is calculated, either by measuring gene expression levels in a mixture of RNA from both parents, as demonstrated in studies using microarray approaches [60,61,62], or, more recently, through RNA-sequencing technology, where reads are counted relative to the expression levels of each parent separately and then averaged [49,50,53]. For example, suppose we have two parental species (A and B) that differ in the expression level of the X gene (10 and 20, respectively). The MPV of the X gene is (10 + 20)/2 = 15. If the allopolyploid derived from the two parental species A and B has an expression of the X gene equal to 15, then we can say that the expression of the X gene is additive in the allopolyploid. The additive condition of parental expression is generally used as reference to compare the overall allopolyploid expression of a gene or a group of genes, i.e., the zero hypothesis. 

If the overall expression of allopolyploid is not additive, then it might be equal to one of the parents (expression level dominance) or it might be lower (transgressive down-regulation) or higher (transgressive up-regulation) than both parental expression levels (Figure 1). In transgressive expression the gene expression is outside the range of the parental expression level. For example, if the expression level of the X gene is 10 and 20 in parental species A and B, respectively, then the expression level of the same gene in allopolyploid is transgressive up-regulated if it is higher than 20, or transgressive down-regulated if it is lower than 10. In practice, differentially expressed genes (e.g., |log2 fold change| ≥ 1 and padj ≤ 0.05) between the allopolyploid (considering the joint expression level of both homoeologs) and its parents are usually binned into 12 conditional states. The first bin divides genes whose expression is not distinguishable from additivity (e.g., padj ≥ 0.05) from those distinguishable from additivity (e.g., padj ≤ 0.05). Genes in the latter group could be further classified into more specific nonadditive states (i.e., expression level dominance and transgressive down/up regulated) [34,35,49,50,53]. According to Rapp et al. [34], the 12 differentially expressed states, distinguished by Roman numbers, are: additive (categories I and XII), expression level dominance (categories II, IV, IX, and XI), and transgressive regulation (categories III, V, VI, VII, VIII, and X) (Figure 1). Genes not differentially expressed between the allopolyploid and the parents are assigned to the “no change” state (Figure 1). It is important to note that the conditions mentioned above can be influenced by various factors, including the specific genes involved, the tissues being analysed, and the environmental conditions [51]. 

## 3. How Allopolyploidy Affects Bioinformatics Transcriptome Analyses

Analysis to estimate gene expression profiles in allopolyploids is challenging, particularly in the steps of read mapping and normalization of gene expression levels. Intrinsic genomic redundancy complicates the analysis, the complexity of which is further amplified in polyploids due to the existence of multiple sets of chromosomes and homoeological variation coexisting with allelic variation. Allopolyploidization leads to genetic, epigenetic, transcriptomic and proteomic variations compared to the parental species, often associated with the activities of repetitive sequences and transposon elements [1,63,64,65]. 

These elements contribute to structural variations in allopolyploid genomes, including insertions, deletions, and rearrangements. These changes create genetic diversity beyond that of the parental species [66,67]. Transposon activity in allopolyploids can alter DNA methylation patterns and histone modifications. These epigenetic changes influence gene expression by regulating chromatin structure and affecting the accessibility of genes for transcription [68,69]. Finally, repetitive sequences and transposons can insert near or within genes, impacting their expression levels. They may also act as regulatory elements or produce small RNAs that modulate gene expression post-transcriptionally [70,71].

As discussed above, in allopolyploids, the relative expression levels of homoeolog gene pairs can be unequal. While, compared to the parental species, the cumulative expression of each homoeolog pair can be additive or not. Moreover, homoeologs can be silenced in a tissue-specific manner, complicating the analysis further [51,72]. Early research, based on the use of microarrays, often failed to distinguish the expression between pairs of homoeolog genes [34,62]. This limitation meant that scientists could only assess the cumulative expression of a target gene, lacking information on the regulation of individual homoeolog copies, i.e., the relative contribution of each homoeolog copy to the overall expression was elusive. Consequently, when comparing gene expression between allopolyploid species and their parents, scientists could only infer whether the combined expression of homoeolog copies was comparable to the MPV or the parents’ expression levels.

High-throughput RNA-sequencing and the availability of bioinformatics and genomic resources (such as the OneKP project, an international initiative that has generated large-scale gene sequencing data for over 1000 phylodiverse plant species [73], and PloiDB, a database of ploidy estimates for seed plants [74]), have significantly improved our ability to define gene expression profiles in allopolyploids and appropriately distinguish the gene expression levels of homoeolog genes. 

Gene expression levels in both allopolyploids and diploid organisms are typically measured using high-throughput RNA-sequencing (bulk RNA-seq). It targets mRNA and/or non-coding RNA libraries for the identification of differentially expressed genes. It aims to estimate the average of global expression values in each sample, elucidating the molecular mechanisms involved in a particular tissue at a given time point. In these experiments, mRNA is extracted, purified, and converted into cDNA. The cDNA is then fragmented and sequenced using next-generation technologies. The resulting sequenced reads are mapped to a reference genome or transcriptome, and the number of reads mapped to each gene (or other biological unit) indicates its expression level. To date, there have been only a few studies that have employed single-cell RNA sequencing (scRNA-seq) technology for analysing allopolyploid transcriptomes. One notable example is the research by Qin et al. [75], which investigated the mechanism of cotton fibre cell initiation from the ovule epidermis in the allopolyploid *G. hirsutum*. 

ScRNA-seq enables profiling of the entire transcriptome of numerous individual cells isolated using microscopic droplets or wells. Molecular barcodes are attached to sequences before amplification to identify the cell origin of each transcript. The core of scRNA-seq analysis is the expression matrix, detailing the transcript counts for each gene and cell, which undergoes data preprocessing and subsequent downstream analyses. Unlike bulk RNA-seq, scRNA-seq provides more precise insights into the heterogeneity of gene expression across different cell types and the molecular trajectory of cell differentiation during development. Nevertheless, bulk RNA-seq remains the primary method for studying expression in polyploids given its cost-effectiveness.

Better identification of homoeologs expression now allows for a deeper understanding of allopolyploidy effects on gene expression and regulation. This includes exploring homoeolog expression bias, dosage compensation, and epigenetic modifications, all of which play a critical role in phenotype variation, environmental responses, and progress in crop improvement. Choosing the best strategy to study the transcriptome of allopolyploids depends on several factors, including the nature of the biological question, the range of bioinformatics tools available, the quantity and divergence of sub-genomes within the allopolyploid, and the available information on the parental species. 

Therefore, it is necessary to consider different analytical approaches in order to optimize the analysis. 

The following paragraphs aim to review the intricate steps and prerequisites involved in transcriptome analysis of allopolyploids, focusing on RNA-seq. These include sub-genome phasing, homoeology inference, RNA-sequencing, read mapping and normalization in the identification of differentially expressed genes.

## 4. Sub-Genome Phasing

In allopolyploid species, multiple parental genomes, called sub-genomes, coexist in a single nucleus. A key step is to assign each homoeolog chromosome and gene copy to the corresponding sub-genome. Notoriously, sub-genome identification is simplified if the genomes of putative progenitors are characterized. Nonetheless, the assignment of homoeologs to the corresponding sub-genomes remains challenging due to divergence between the parental and allopolyploid genomes, the high similarity between sub-genomes, and the reshuffling events that characterize the allopolyploid genome. This reorganization includes phenomena such as the loss of chromosomes, homoeologs, and duplicated genes, known as “fractionation” [76,77,78,79]. Fractionation, if it affects the entire polyploid genome, contributes to “diploidization”. Homoeolog exchanges represent another aspect of this dynamic process, which involves the mispairing of chromosomes of different sub-genomes [80,81,82,83]. Because of these exchanges, DNA fragments of varying sizes are swapped between sub-genomes, potentially leading to deletions, duplications, and translocations. Although most recombinations between sub-genomes occur between homoeolog chromosomes, occasionally they also occur between non-homoeolog regions [82]. 

The identification of sub-genomes is often necessary as preparatory information for homoeolog annotation and correctly distinguishing them from paralogues and allelic variants. Additionally, this identification enables the analysis of differential expression between sub-genomes, offering insights into their distinct regulatory mechanisms and enhancing our understanding of the biology and evolution of the studied organism. 

Sub-genome inference is achieved using supervised and unsupervised bioinformatics approaches. The supervised approach exploits data and sequences from diploid parents or closely related species. When such information is available, sub-genomes can be distinguished by mapping allopolyploid-derived DNA- or RNA-seq data on one or both reference genomes, taking advantage of sequence similarity or homeolog-specific polymorphisms [84,85,86]. To achieve this, specialized mapping and categorization algorithms designed specifically for allopolyploid organisms can be used, including PolyCat [87], SNiPloid [84], and HANDS2 [88], particularly if a single reference genome is accessible. For example, Kim et al. [89] followed the PolyCat pipeline, aligning reads to the *Gossipium raimondii* reference genome (the closest extant relative of *G. hirsutum*) in order to identify the sub-genome of *G. hirsutum*.

Alternatively, PolyDog [90] is used when both parental genomes are available (more details about mapping and categorization algorithms will be given in the subsequent paragraph). Further evidence to support sub-genome assignment can be obtained from multi-locus synthetic phylogenetic trees [43] and synonymous nucleotide substitution approaches implemented in the WGDI pipeline [91]. Sun et al. [92] employed the WGDI pipeline to explore the evolutionary history of the cotton family using seven distinct reference genomes. Their approach involved extracting the proto-chromosomes of extant species to reconstruct the detailed karyotype evolution of the family. This was achieved through the detection of collinear genes, estimation of synonymous substitution rates (Ks), and the construction of synteny trees.

When both the sub-genomes and the parental species genomes have been diverging for a long period of time, the sub-genomes are not easily distinguishable. To overcome this hurdle, the sub-genomes separation can be based on large phylogenetic data collection, as demonstrated by Schiavinato et al. [93]. Specifically, by evaluating the phylogenies of all individual genes within the allopolyploid species and their homologs in the parental species, it becomes feasible to rank parental leaves based on distance. This process allows for the assignment of parental origins to each homoeolog gene pairs by selecting the leaf with the shortest phylogenetic distance.

The unsupervised strategy comes into play when diploid ancestors are unknown and the methods rely on differences in sub-genome properties, such as GC content or the presence of sub-genome-specific repetitive sequences (e.g., transposable elements). Various programs, including PolyCRACKER [94] and SubPhaser [95], implement this methodology and require chromosome-scale genome assembly. Among scientists using unsupervised approaches, Song et al. [96] assembled a gap-free genome for the cultivated octoploid strawberry “Benihoppe” and phased its sub-genomes. They identified sub-genome-specific sequences (k-mers) to assign homoeologous chromosomes to their respective sub-genomes using the SubPhaser algorithm. Recently, Zhang et al. [97] provided a valuable benchmark and recommendations for assessing the accuracy of the WGDI and SubPhaser tools. Additionally, a comprehensive review on allopolyploid sub-genome identification was conducted by Session [98].

## 5. Homoeology Inference

The identification of homoeologs in transcriptome analysis is essential for obtaining a detailed and comprehensive view of gene expression. It helps determine whether varying expression levels of homoeologs across different sub-genomes influence the phenotype and reveals whether homoeologs have retained similar functions (sub-functionalization) or acquired new functions (neo-functionalization).

The process of identifying ortholog genes, i.e., genes derived from a single ancestral gene and now present in different species [99], overlaps in some ways with the identification of homoeologs in allopolyploids [25]. Indeed, homoeologs within the sub-genomes of an allopolyploid species could be considered as orthologs between distinct species, given their common origin from speciation events. Their identification does not imply that the genes have the same function but rather that they arise from a speciation event [99]. Nevertheless, in comparative genomics the “ortholog conjecture” states that ortholog genes, and therefore also homeologs, have similar functions [100]. In comparative genomics, homologs genes resulting from a gene duplication event are called paralogs, and those from recent duplications can maintain similar functions compared to distant paralogs separated by millions of years of evolution [101]. Paralogs that resulted from a duplication event that occurred before speciation are called outparalogs, while those that occurred from a post-speciation duplication are called inparalogs [102]. Since inparalogs can be grouped under a single gene and defined as co-orthologs (“orthologs group”), not all orthologs in the same group arise from speciation. This situation could be considered a “terminology muddle”, as defined by Ouzounis et al. [103], since the term extends the relationship to include inparalogs relative to another gene in a different species [104]. In the context of a hybridization event, these genes would be classified as homoeologs to each other [105]. According to this classification method, homoeolog relationships could vary from one-to-one, one-to-many, or many-to-many, depending on the number of duplications since the speciation of the progenitors [25]. It is important to note that in allopolyploids and polyploids in general, the presence of redundant genes as paralogs allows the latter to acquire, relatively quickly, mutations, leading to the emergence of sub- neo- and non-functionalizations [7]. Outparalogs, having had more time to evolve and potentially change function compared to inparalogs, are typically not considered co-orthologs [106]. Similarly, they should not be classified as homoeologs for the same reason. 

Programs developed to identify orthologs could be adapted to infer homoeologs in allopolyploid species, given the parallels between the two. This often requires a priori knowledge of sub-genomes in allopolyploids. Bioinformatics inference of homoeologs involves a combination of computational and analytical approaches. The choice of method depends on the specific requirements of the study and the available resources. Various programs have been developed for this purpose; these can be mainly divided into graph-based and tree-based approaches. The graph-based method assumes that orthologs or homoeologs share sequence similarities. The graph construction phase identifies orthologs or homoeologs through similarity searches, considering pairs of genomes or sub-genomes at a time. In these graphs, genes (or proteins) are depicted as nodes, and their evolutionary relationships are represented as edges [107].

The Bidirectional Best Hits (BBH) approach is the simplest and most widely used method based on sequence similarity [108]. This method assumes that two genes are homoeologs if each gene is the best match of the other when their respective sub-genomes are compared. In simpler terms, for a gene pair to be classified as homoeologs, each gene must show higher similarity (e.g., highest alignment score) to its pair than to all other genes in the other sub-genome [109]. Similarity is typically assessed using BLAST [110] or other sequence alignment algorithms. However, the BBH method has a limitation: it can only identify orthology in a 1-to-1 relationship [111]. To complement this method, synteny (the conservation of gene order between two chromosomal regions [112]) is often employed as additional evidence for identifying homoeologs [113]. Even when both methods are used, the ability to detect genes that have duplicated and relocated, both before and after allopolyploidization events, remains a challenge. For instance, in plants where gene duplication rates are high, the BBH method alone may fail to identify up to 60% of orthologous relationships in datasets involving 12 Viridiplantae species [111]. Even when BBH and synteny methods are combined, they may still fail to detect up to 26% of homoeologs relationships in cotton [109]. To address this limitation, other algorithms such as OMA [114] and OrthoFinder [115] have been developed to incorporate inparalog relationships into the graph construction process. The OMA pipeline has been adapted to infer homoeologs in recent studies [105]. In these studies, homoeologs were inferred in three polyploid species: upland cotton (*G. hirsutum*), rapeseed (*B. napus*), and bread wheat (*T. aestivum*). Each sub-genome was treated as a separate genome, and orthologs were inferred using the standard OMA pipeline [114]. Similarly, Zhang et al. [116] identified homoeologs in a synthetic allotetraploid wheat resulting from a cross between the diploid parent species *Triticum urartu* (AA) and *Aegilops tauschii* (DD). They inferred orthologs between parental genomes using OrthoFinder. 

Tree-based methods rely on the assumption that genes share an evolutionary history and employ a resolution technique known as “tree reconciliation”. In this approach, the structure of a gene tree is compared with that of a species tree, and they are reconciled based on the principle of parsimony, aiming to infer the least number of duplication and loss events during the gene’s evolution [106]. To begin this method, a multiple alignment of homologous sequences is first generated, which forms the basis for constructing a phylogenetic tree of the gene family. During the reconciliation phase, the nodes of this gene tree are classified as either duplication or speciation events by comparing them with the nodes of the species tree. This method is implemented in various software, including Ensembl Compara [117]. The Ensembl Compara pipeline utilizes the TreeBeST algorithm (http://treesoft.sourceforge.net/treebest.shtml, accessed 1 July 2024), originally developed for TreeFam [118], to infer reconciled gene trees. This pipeline was utilized by Xu et al. [119] to infer orthologous genes for studying the evolutionary history of the polyploid family Cyprininae.

The effectiveness of tree-based methods hinges heavily on the accurate construction of multiple alignments and trees, which can be computationally intensive and thus limit their application to large datasets [101]. A hybrid approach that integrates graph- and tree-based methods has been implemented in HomoloGene. In contrast, other software employs a meta-approach to leverage predictions generated by multiple programs, such as MARIO [120] and MetaPhOrs 2.0 [121]. These methods have been extensively reviewed in several studies [25,101,106,109,122]. It is important to note that when inferring homoeologs based on RNA-seq-derived sequences, misidentification may occur if one or both homoeolog genes are not expressed.

## 6. RNA-Sequencing

In recent years, expression profiling in allopolyploid species has commonly involved the analysis of RNA-seq data obtained through next-generation sequencing (NGS) technology, with a notable preference for short-read sequencing. Despite the availability of several NGS technology providers, these short reads are often generated using Illumina instruments due to lower cost, high throughput and availability for a wide range of tools and pipelines also suitable for polyploid species [123,124]. For example, through the Illumina HiSeq 2000 platform, Peng et al. [52] studied the expression patterns of homolog genes between the allotetraploid cotton *G. hirsutum* and its diploid progenitors *G. arboreum* and *G. raimondii* at the early fibre development stage. While, through Illumina NextSeq 500, Gault et al. [125] performed a de novo transcriptome assembly of the tetraploid species *Tripsacum dactyloides*, a sister genus of *Zea mais*, in order to demonstrate that similar genes may be decaying into pseudogenes in both genera after a shared ancient polyploidy event. Considering that the cost of sequencing a genome is affected by its size, the cost for polyploid organisms could be double that of their diploid counterparts. Recently, Sun et al. [33] explored the expression of homoeologs in allohexaploid wheat and demonstrated cost-effective sequencing using 3′ UTR RNA-seq with the Lasy-Seq protocol [126]. However, using short reads poses challenges in transcriptome assembly and isoform identification, especially in allopolyploids where homoeolog genes often produce highly similar isoforms [127]. These challenges are typically addressed by leveraging RNA-seq long reads. Long-read sequencing technologies such as PacBio Iso-Seq [128] and Nanopore-Based Direct RNA sequencing [129] offer improvements in de novo assembly, mapping accuracy, identification of transcript isoforms, and detection of structural variants. Moreover, direct RNA sequencing from native molecules eliminates amplification biases while preserving base modifications [130]. Following this approach, Wang et al. [127] used long-read sequencing to identify approximately 175,000 isoforms in nearly 45,000 genes in allotetraploid cotton, revealing that approximately 50% of homoeolog genes produce divergent isoforms in each sub-genome. Consistent with these findings, Wang et al. [131] subsequently identified numerous novel transcripts in response to salt stress. Additionally, Yao et al. [132] utilizing PacBio Iso-Seq, performed a comprehensive transcriptome analysis of the allopolyploid *B. napus*, leading to the construction of an isoform sequencing database.

## 7. Mapping Reads

Transcriptome analysis in allopolyploids can produce two types of valuable data. First, uniquely mapped reads can reveal the expression levels of homoeologs, provided there are sufficient polymorphisms between homoeolog sequences. Second, ambiguously mapped reads can indicate the absolute expression abundance for each pair of homoeologs. The mapping process can use the parental genomes as references, treating homoeologs as orthologs [27], or it can be based on the assembled genome of the allopolyploid itself. 

Assembling an allopolyploid genome presents significant challenges, exacerbating the difficulties already encountered in diploid genome assembly. One major issue is the incorrect assembly of fragments from one sub-genome into another, leading to chimeric and fragmented contigs. This challenge is primarily due to repetitive sequences, such as satellite DNA, DNA transposons, retrotransposons and variation in gene copy-number. These repetitive elements are prevalent in allopolyploid genomes, and their accurate detection is essential for correct genome annotation [133,134,135]. 

It is important to note that using reference sequences discordantly annotated can distort the assessment of differential expression of homoeologs. This issue is particularly significant because annotations, often based on RNA-seq data, may incompletely or incorrectly annotate homoeolog exons, especially for genes expressed at low levels. Consequently, depending on the tool, the mapping stage may be restricted to accurately annotated loci in both sub-genomes. 

In the absence of a reference genome, de novo assembly of the transcriptome involves reconstructing the transcript set from multiple samples, potentially under varying conditions, to achieve a transcriptome as comprehensive as possible. However, assembling the transcriptome of an allopolyploid presents nearly two additional challenges compared to its diploid parental species. The first challenge, known as “homoeologs collapse”, occurs when two homoeologs are erroneously assembled into a single hybrid transcript. The second challenge, referred to as “SNP shuffling”, arises when a homoeolog is assembled incorporating polymorphisms from the other homoeolog, complicating accurate sequence reconstruction [136]. In cases where parental transcriptomes or genomes need to be assembled, accurately identifying the parents is crucial but not always straightforward. This complexity underscores the meticulousness required in transcriptome assembly of allopolyploids to avoid these pitfalls and ensure robust data interpretation.

Once reference sequences are identified, two distinct mapping approaches can be used. The first approach utilizes traditional algorithms originally developed for diploid organisms. Well-known tools, such as STAR [137] and HISAT [138], are used to align RNA-seq reads directly to the reference genome. Several studies have utilized such algorithms. For example, Burns et al. [139] used STAR to map RNA-seq reads to a de novo assembled genome of the allopolyploid *Arabidopsis suecica*. Meanwhile, Wu et al. [50] employed HISAT2 to explore global gene expression patterns in resynthesized allotetraploid *B. napus.* Additionally, pseudoalignment programs like Kallisto [140] provide alternative methods for efficient sequence alignment based on transcript abundance estimation. The second approach involves specialized pipelines specifically designed for polyploid organisms. These methods typically fall into two categories:

- Similarity-based mapping: These pipelines compare allopolyploid reads to loci in the genomes of the parental species to identify homoeolog sequences.
- Genotype-aware mapping: These pipelines aim to detect genotypic differences and classify allopolyploids reads to the corresponding sub-genomes.

Both approaches play crucial roles in overcoming the challenges unique to allopolyploid transcriptome analysis, ensuring accurate quantification of gene expression and facilitating comprehensive understanding of gene regulation in allopolyploid organisms. As a result, these pipelines generate sub-genome specific BAM files that serve as inputs for downstream bioinformatics analyses. Several tools leverage similarity searches for such analyses, including HomeoRoq [141] and PolyDog. The HomeoRoq pipeline requires genomic sequences from both parental organisms and employs STAR to map RNA-seq reads to each reference sequence independently. Reads are then classified based on mismatch counts to determine their genomic origin. 

Recently, Sun et al. [142] utilized the HomeoRoq pipeline to classify the genomic origins of RNA-Seq reads in the allotriploid *Cardamine insueta*. They achieved a remarkably low percentage of mismapping, with only 1.1 ± 0.1% in *Cardamine amara* and 1.2 ± 0.1% in *Cardamine rivularis*. 

Similarly, PolyDog uses genomic sequences from both parental genomes as references. It employs GSNAP [143] as its default mapping tool to align RNA-seq reads. The final assignment of reads to specific sub-genomes is determined by considering quality scores, alignment length, and mismatch quantity.

Several tools utilize genotypic differences to classify RNA-seq reads to their respective sub-genomes in allopolyploid organisms. Among these tools are PolyCat and EAGLE-RC [144]. PolyCat uses a single reference sequence along with GSNAP as its default mapping tool. It utilizes SAMtools [145] for SNP calling to identify “homoeo-SNPs” between parental diploid reads and the reference genome. PolyCat then generates a SNP index, which is used to categorize allopolyploid reads into their respective sub-genomes based on matches with identified homoeo-SNPs. The approach described was utilized by Dong et al. [146], who investigated changes in genic coexpression resulting from the domestication of *G. hirsutum*. They classified RNA-Seq reads from the allotetraploids *G. hirsutum* var. TM1 and *Gossypium tomentosum* using the reference genome of *G. raimondii*. This process involved SNP-tolerant mapping between the genomes of the diploid parents *G. arboreum* and *G. raimondii*. 

Similarly, EAGLE-RC utilizes an alignment in BAM format and a set of variants in VCF format, along with a single reference genome. It classifies reads based on genotype differences. EAGLE-RC also offers a mode (--ngi mode) that directly classifies reads using the likelihood derived from alignments of different sub-genomes, without explicit genotype information. Recently, Katayama et al., 2024 [147] employed EAGLE-RC to determine the parental origin of the reads in the allotetraploid *Phegopteris decursivepinnata*. They used two BAM files generated from mapping of the RNA-seq reads to the reference gene sets of *Phegopteris koreana* and *Phegopteris taiwaniana*.

When aiming to determine homoeolog expression levels, benchmark studies have demonstrated that traditional software originally designed for diploid species results in higher error rates, particularly exceeding 10% when using pseudoalignment algorithms. However, using sub-genome classification pipelines can significantly reduce mapping errors. For instance, tools like EAGLE-RC achieve error rates below 1%, while HomeoRoq typically achieves error rates less than 2%, depending on variables such as species, expression level, and read length. It is crucial to note that discrepancies in expression level assessment primarily stem from genes with low expression levels. These genes can cause significant shifts in the calculation of expression ratios between homoeologs [30].

## 8. Normalization in Differential Expression Analysis

Transcriptome profiling is commonly applied to identify differentially expressed genes. In differential expression analysis, high-throughput RNA-seq data is used to determine if there are statistically significant differences in read abundance at each locus across different experimental conditions. This analysis can involve comparing homoeolog genes within the allopolyploid genome or assessing the global expression levels of each sub-genome relative to their respective parental genomes. These comparisons are crucial for addressing diverse biological questions, including identifying and understanding the mechanisms that enable polyploids exhibiting superior vegetative growth compared to their progenitor species. 

Before identifying differentially expressed genes, it is crucial to address biases by normalizing read counts obtained during the quantification step. Errors in normalization can lead to significant consequences in downstream analysis, such as an increased rate of false positives in differential expression analysis [148]. The primary goal of normalization is to mitigate biases so that differences in normalized read counts accurately reflect true differences in gene expression between samples. Thus, a gene is deemed differentially expressed across biological conditions if it shows varying mRNA levels per sample under those conditions [149]. The biases that mainly impact the detection of differential expression include gene length, GC content (the proportion of guanine and cytosine nucleotides), and library size (total number of sequenced reads per sample, i.e., sequencing depth). Gene length and GC content primarily introduce biases in comparisons of gene counts within a single sample (“within-sample” bias). These factors are often disregarded in differential expression analysis between two or more biological conditions. However, they can affect the accuracy of expression level quantification if not appropriately normalized or accounted for. On the other hand, library size plays a crucial role in comparisons of the same gene’s counts across different samples (“between-sample” bias). Differences in sequencing depth between samples can lead to misleading conclusions about differential expression if not adjusted during normalization.

In RNA-seq libraries, read counts directly correspond to relative gene abundances, meaning highly expressed genes can dominate, thereby reducing the number of reads available for less expressed genes. Consequently, increasing library size enhances the detection sensitivity for poorly expressed genes. Normalizing samples with different library sizes is crucial because without normalization, a sample sequenced at a higher depth than another would show inflated read counts for all genes. This could potentially lead to erroneous conclusions regarding differential expression between samples. 

The simplest method of between-sample normalization involves scaling raw read counts within each sample by a sample-specific factor that reflects its library size [149]. Several data normalization methods have been developed to mitigate this bias in RNA-seq analysis. FPKM (fragments per kilobase million) [150] and TPM (transcript per million) methods [151] primarily focus on “within-sample” normalization, adjusting for gene length within individual samples [152]. In contrast, TMM (edgeR’s Trimmed Mean of M values) [153] and RLE (DESeq’s relative log expression) [154], are applied for “between-sample” normalization where differences in sequencing depth between samples are addressed. 

No normalization method is without limitations, and each approach has scenarios where its assumptions may not hold. For instance, methods like TMM and RLE are effective in managing differences in mRNA levels per cell across conditions when there are only a few differentially expressed genes, particularly in cases with minimal asymmetry among highly expressed genes. However, these methods may struggle to handle situations involving a global shift in gene expression, such as when there is a proportional increase in mRNAs per cell due to changes in ploidy levels. This scenario can lead to substantial asymmetry in the number of highly expressed genes between conditions [149]. Studies have highlighted instances where varying ploidy levels can cause significant global shifts in gene expression, thereby challenging the applicability of traditional normalization methods [155,156,157].

Therefore, the choice of a normalization strategy depends on the specific biological question being addressed and the characteristics of the samples under study. For example, when comparing samples from the same cell type in diploid organisms, the number of mRNA molecules per cell (transcriptome size) typically remains relatively stable. However, this consistency does not hold when comparing samples with different ploidy levels, as observed in [156,158,159]. This is because polyploid organisms often exhibit larger cells, which are positively correlated with increased transcriptome size [160,161]. 

Traditional sample preparation methods that solely standardize by sample concentration, without accounting for biomass or cell count, may result in inaccurate interpretations [162]. For instance, when assessing whether changes in gene expression correlate with variations in photosynthesis per unit of surface area, it can be more insightful to compare expression levels among samples with equal biomass quantities. This approach provides a more robust basis for analysing gene expression patterns than relying solely on sample concentration-standardized preparations [159]. Therefore, particularly when comparing organisms with different ploidy levels, it is crucial to consider various scales of comparison: per transcriptome (RNA concentration-based), per cell, and per biomass (surface area or weights) [159,162]. Using these different approaches, studies have demonstrated that the polyploid plant *Tolmiea menziesii* (which has a lower cell density compared to the diploid *Tolmiea diplomenziesii*) tends to maintain diploid-like transcript abundance per biomass but increasing gene expression per cell. Notably, this enhanced expression is enriched for functions related to photosynthesis [159].

To address normalization issues related to biomass or cell count and mitigate transcription size bias, scientists have proposed the use of multiple different exogenous “RNA spike-ins” [163]. These RNA spike-ins, known for their precise concentrations, are added in proportion to the biomass or number of cells prior to RNA-seq library preparation. Subsequently, these spike-ins serve as scaling factors for between-sample normalization after quantification. Implementing RNA spike-ins requires prior knowledge of the biomass quantity or the number of cells included in each sample for accurate normalization. When direct cell counting is impractical, scientists can estimate cell numbers based on DNA content using techniques such as flow cytometry [157,163].

In the context of the scenarios mentioned earlier, analysing differentially expressed genes in allopolyploids can present more complexity compared to standard RNA-seq analyses. A first approach to analysis was introduced by Rapp et al. [34], working on allotetraploid cotton *Gossypium* L. genus. They performed comprehensive comparisons including relative expression levels between the two parents, the hybrid allopolyploid versus its parents, and the allopolyploid versus the mid-parent value. From these comparisons, Rapp et al. [34] categorized allopolyploid expression patterns into 12 distinct categories (as detailed in the “Terminology in allopolyploid gene expression” section). This method has since been adopted by numerous subsequent studies [49,52,164,165,166,167].

Recently, Almeida-Silva et al. [168] adopted a similar strategy, utilizing RNA-sequencing data to explore the transcriptional responses to saline stress in cotton and heterosis events in root traits in rice. In their studies, they introduced HYBRIDEXPRESS, a tool designed to facilitate comparative analyses between allopolyploid hybrids and parental lines. This tool encompasses transcriptomic data processing, graphic visualization, data normalization, identification of differentially expressed genes, classification of genes based on the stages proposed by Rapp et al. [34], and functional annotation of these genes. Such tools, alongside advancements in sequencing technologies and methodologies for homoeology inference and sub-genome identification, hold significant promise in elucidating parental legacies, homoeolog expression biases in allopolyploids, understanding their evolutionary mechanisms, and providing crucial insights for crop breeding.

## 9. Conclusions

Transcriptome analysis in allopolyploid plants is crucial for unravelling the complexities of gene expression resulting from genome duplication through hybridization. This process not only sheds light on the evolution of duplicated genes and their adaptive potential in new environments but also reveals novel genes and regulatory mechanisms important for agricultural and biotechnological advancements. However, conducting such analysis poses significant bioinformatics challenges due to the presence of highly similar homoeolog genes and the need for precise methods in read mapping, assembly, and expression quantification. Overcoming these challenges requires meticulous experimental design and the application of advanced analytic strategies to ensure accurate interpretation of the transcriptomic data. Despite these hurdles, the insights gained from transcriptome studies in allopolyploid plants promise to deepen our understanding of plant evolution and facilitate targeted improvements in crop breeding and genetic engineering.

## Data Availability

Not applicable.
